# An open relaxation-diffusion MRI dataset in neurosurgical studies

**DOI:** 10.1038/s41597-024-03013-9

**Published:** 2024-02-07

**Authors:** Ye Wu, Xiaoming Liu, Yunzhi Huang, Tao Zhou, Fan Zhang

**Affiliations:** 1 School of Computer Science and Technology, Nanjing University of Science and Technology, Nanjing, China; 2 Department of Radiology, Union Hospital, Tongji Medical College, Huazhong University of Science and Technology, Wuhan, China; 3 Hubei Province Key Laboratory of Molecular Imaging, Wuhan, China; 4 School of Artificial Intelligence, Nanjing University of Information Science and Technology, Nanjing, China; 5 School of Information and Communication Engineering, University of Electronic Science and Technology of China, Chengdu, China

**Keywords:** Biophysical models, Tumour heterogeneity

## Abstract

Diffusion MRI (dMRI) is a safe and noninvasive technique that provides insight into the microarchitecture of brain tissue. Relaxation-diffusion MRI (rdMRI) is an extension of traditional dMRI that captures diffusion imaging data at multiple TEs to detect tissue heterogeneity between relaxation and diffusivity. rdMRI has great potential in neurosurgical research including brain tumor grading and treatment response evaluation. However, the lack of available data has limited the exploration of rdMRI in clinical settings. To address this, we are sharing a high-quality rdMRI dataset from 18 neurosurgical patients with different types of lesions, as well as two healthy individuals as controls. The rdMRI data was acquired using 7 TEs, where at each TE multi-shell dMRI with high spatial and angular resolutions is obtained at each TE. Each rdMRI scan underwent thorough artifact and distortion corrections using a specially designed processing pipeline. The dataset’s quality was assessed using standard practices, including quality control and assurance. This resource is a valuable addition to neurosurgical studies, and all data are openly accessible.

## Background & Summary

Diffusion magnetic resonance imaging (dMRI) is a noninvasive imaging technique that can probe the diffusion of water molecules in biological tissues to characterize the underlying microstructure^1,2^. There have been many methods proposed for extracting tissue microstructure and measuring from the dMRI signals using either signal representation or tissue modeling approaches^2–6^. Most of the existing methods use standard dMRI data that is acquired with a single echo time (TE) and thus provide information primarily on diffusivity. Recently, studies have shown that integrating dMRI with multiple TEs can better characterize tissue microstructure than dMRI with a single TE^7–11^.

Relaxation-diffusion MRI (rdMRI) is an advanced technique that combines measurements of tissue relaxation times (T1 and T2) and diffusion imaging to investigate the microstructural and physiological characteristics of tissues^12–15^. In rdMRI, transverse relaxation refers to the time that it takes for the transverse magnetization of a tissue to decay after an excitation pulse. Combining with diffusion imaging, transverse relaxation time is a crucial imaging parameter to study tumor characteristics, including tumor grading, edema assessment, necrotic areas, and treatment response assessment^16–19^. Table 1 gives a summary of potential neurosurgical applications that can benefit from using rdMRI.

**Table 1:** Transverse relaxation time provides valuable information about tumor characteristics.

Applications	dMRI	rdMRI
Tumor Grading	Low-grade gliomas typically exhibit higher apparent diffusion coefficient (ADC) values compared to high-grade gliomas^35,36^. This is due to the presence of less densely packed tumor cells and less restrictive tissue structures, allowing for greater water diffusion.	Due to increased cellularity, nuclear-to-cytoplasmic ratio, and necrotic areas, higher-grade gliomas often exhibit shorter transverse relaxation times than lower-grade gliomas^37–40^ Thus, rdMRI can be a useful technique for grading different gliomas.
Edema Assessment	In areas of edema, there is typically an increase in extracellular fluid and disruption of tissue architecture, leading to changes in water diffusion^41,42^. The ADC values in regions affected by edema are generally higher compared to normal tissue because the restricted diffusion barriers are disrupted, allowing for increased water movement.	Edema around a glioma is associated with malignancy of the tumor. Transverse relaxation time maps can help visualize and quantify peritumoral edema^43,44^. The extent of T2 hyperintensity surrounding the tumor reflects the degree of edema, which can influence treatment decisions and surgical planning.
Necrotic Areas	dMRI reflects the mobility of water molecules within tissues. In necrotic areas, there is a disruption of tissue architecture, loss of cell membrane integrity, and breakdown of cellular components^45,46^. These changes result in increased extracellular space, reduced cellular density, and altered water diffusion patterns.	High-grade gliomas often contain necrotic regions. Necrotic areas typically have longer transverse relaxation times than solid tumor regions, reflecting the increased water content and altered tissue composition^47,48^. In this way, rdMRI can help identify and evaluate the presence and extent of necrosis within a glioma.
Treatment Response Assessment	During successful treatment, there are often changes in tumor biology and tissue architecture that can be reflected in dMRI-derived microstructure values. Generally, an effective treatment leads to decreased cellularity, increased extracellular space, and improved tissue organization^49–52^. These changes result in increased water diffusion and higher neurite-related values within the treated region.	Transverse relaxation time can also be used to monitor treatment response in gliomas. Changes in transverse relaxation time throughout treatment can indicate alterations in tumor characteristics^53–55^, such as reduction in edema or necrosis, response to therapy, or recurrence. Serial transverse relaxation time measurements provide valuable information for assessing treatment efficacy and guiding further management decisions.

The development of relaxation-diffusion imaging with multi-echo is still evolving, challenged by the lack of effective acquisition protocols and robust data processing and analysis methods. These efforts promise to improve its clinical applicability and enhance our understanding of glioma biology and treatment response. rdMRI^7–11^ acquired diffusion signals at different TEs following diffusion sensitizing gradients, which have some challenges. First, acquiring multiple echoes requires additional scan time, which can limit its application in specific clinical settings or when fast imaging is necessary. Second, rdMRI data with multi-echo requires more complex processing and analysis techniques than single-echo dMRI. Separating signal contributions from different compartments and estimating microstructure parameters involve advanced modeling and fitting algorithms. Third, rdMRI can be more sensitive to susceptibility-induced artifacts due to the longer echo time. This can lead to distortions and signal loss, particularly in regions prone to susceptibility effects, such as the frontal lobes or areas near air-tissue interfaces. Currently, no open rdMRI datasets are available for broad scientific and clinical investigations.

Given the above, we disseminate a dataset of rdMRI scans acquired at 3 T. We make available a high-quality rdMRI dataset from a cohort of 18 neurosurgical patients with different types of lesions, plus two healthy individuals as controls. We comprehensively describe the dataset’s design, acquisition, and preparation. The rdMRI data is acquired on a 3 T Philips MRI scanner with 7 TEs. At each TE, multi-shell diffusion-weighted images with high spatial and angular resolutions are acquired. Each rdMRI scan is well processed for artifact and distortion corrections using a newly designed rdMRI-specific processing pipeline. Image quality is assessed using quality metrics implemented in multiple popular tools. Using this new resource, we also provide codes, preliminary results, and perspectives for future projects. Constituting an essential new resource for neurosurgical studies, all data are openly available. We expect this dataset to serve as a valuable resource for refining acquisition techniques, optimizing acquisition parameters, and establishing standardized approaches for rdMRI in studying gliomas.

## Methods

### Participants

The MRI data was collected from 18 patients (including glioma, meningioma, diffuse large B-cell, multiple sclerosis, cortical cerebral infarction, and brain abscess) and two healthy individuals (11 females and 9 males; age range: 28.0–70.0 years; median age: 51.0 years; IQR: 21.5 years). All participants provided written informed consent before participation and signed informed consent regarding publishing their data. The Research Ethics Committee, Faculty of Medicine in Union Hospital, Tongji Medical College, Huazhong University of Science and Technology, China, approved the study protocols (approve number: 2021-IEC-0984).

### Image acquisitions

All MRI scans were acquired using a Philips 3 T MRI (Ingenia CX, Netherlands) scanner with a gradient strength of 80 mT/m and switching rates of 200 mT/m/msec, equipped with 32-channel head coils.

**Fig. 1:**
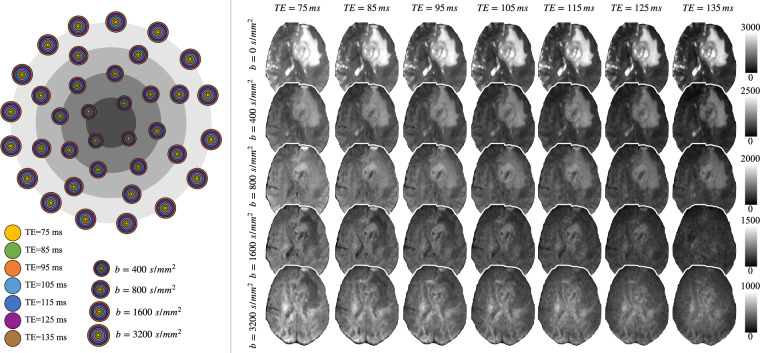
Multi-shell, multi-echo dMRI acquisition, and the example rdMRI data from a high-grade glioma patient.

The rdMRI data is scanned using a multi-shell, multi-echo dMRI sequence (Fig. 1) with a fixed repetition time (TR) = 4000 ms, {4, 8, 8, 16} noncollinear diffusion-encoding directions at each of four *b* = {400, 800, 1600, 3200}s/mm^2^ respectively, Δ = {35.9, 40.9, 45.9, 50.9, 55.9, 60.9, 65.9}ms, *δ* = {19.9, 24.9, 29.9, 34.9, 39.9, 44.9, 49.9}ms, echo times: *TE* = {75, 85, 95, 105, 115, 125, 135}ms, 1.5 mm isotropic voxel size. Together with four acquisitions without diffusion weighting (*b* = 0 s/mm^2^); the number of averages = 1; field of view = 160 mm × 160 mm; matrix = 130 × 130; flip angle = 90; 96 axial slices with gap = 0; multi-band factor = 4. These diffusion data were obtained using a spin-echo echo-planar imaging sequence.

In addition, T1-weighted three-dimensional (3D) turbo field-echo (TFE) parameters include 250 sagittal 1 mm slices; 1 mm isotropic; 250 × 250 matrix; repetition time/echo time (TR/TE) = 8.568/4.154 ms; flip angle = 8. T2-FLAIR was also acquired with the parameters including 250 sagittal 1 mm slices; 1 mm isotropic; 250 × 250 matrix; repetition time/echo time (TR/TE) = 4800/306.987 ms; flip angle = 90.

### Image processing

The acquired MRI scans were converted from DICOM to Neuroinformatics Informatics Technology Initiative (NIfTI) format using dcm2niix in MRIcroGL (v1.2) (https://www.nitrc.org/plugins/mwiki/index.php/mricrogl) and then organized following the Brain Imaging Data Structure (BIDS) format^20^. Facial information was removed from all the MRI scans using PyDeface (v2.0.2) (https://github.com/poldracklab/pydeface). MRI image was reoriented using ‘fslreorient2std’ in the Functional Magnetic Resonance Imaging of the Brain (FMRIB) Software Library tool (FSL v6.0.3)^21^ to match the approximate orientation of the standard template images, and the axis-aligned and centered using pnlNipype^22^ to ensure non-diagonal alignment in the affine transform.

**Fig. 2:**
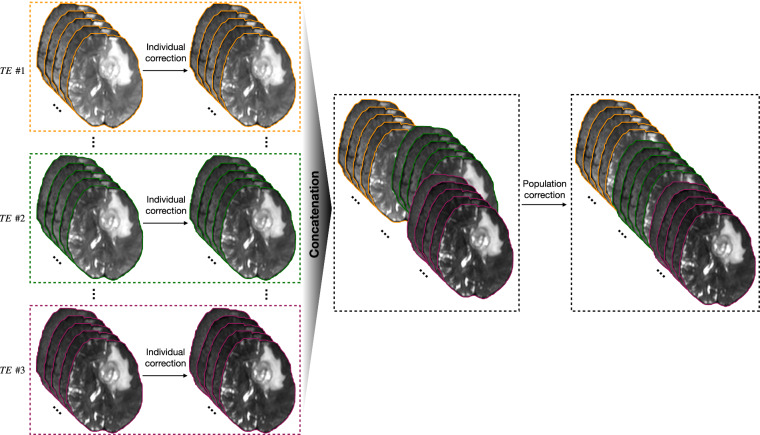
Preprocessing pipeline consists of individual and population correction for MRI data.

The preprocessing pipeline for diffusion-weighted images (DWI) was performed separately for each TE session, as shown in Fig. 2. This involved denoising using MRtrix3^23^, correcting for eddy current-induced distortion, motion, and bias field using FSL^21^.

To make sure the gradient table is in the correct order, we used the following two strategies, as reported by Cai *et al*.^24^ and Snoek *et al*.^25^. Firstly, we exported the actual gradient table from the DICOM using the latest version of dcm2niix, instead of using the predefined gradient table. This ensured that the corrected ordering corresponded with the DWI volume and that all gradients had been reoriented into subject space without any reordering of image volumes. Secondly, we used ‘dwigradcheck’ in MRtrix3 to correct any possible issues with the diffusion gradient table further.

Next, for each subject, to align the DWIs across multiple TE sessions (Fig. 2), a joint eddy current-induced distortion correction and motion correction were performed in concatenated DWIs over all of the TE sessions. To further correct for distortions caused by magnetic field inhomogeneity, an EPI distortion correction was conducted regarding the T1-weighted image using Advanced Normalization Tools (ANTs v2.5.0)^26^. To this end, we first generated a T2-weighted-like contrast image from a T1-weighted image using in-house software. For each participant, a nonlinear registration (restricted to the phase-encode direction) was computed from the b0 image to the synthetic T2-weighted image to make an EPI corrective warp. Then, the warp was applied to each DWI, followed by reorientation of the corresponding gradient directions. Each individual’s T1-weighted image was also transformed from structural space into diffusion space via rigid registration using FSL^21^. Finally, processed DWIs were split into the corresponding TE session.

The preprocessing pipeline for T1-weighted and FLAIR images consisting of Gibbs ringing artifact removal and bias field correction was performed using ANTs^26^. Brain masks were created using a Convolutional Neural Network (CNN) based segmentation tool in pnlNipype^22^.

## Data Records

Our neuroimaging dataset is now available in two formats: raw and preprocessed MRI data, both of which are available for all release subjects. Demographic information can be found in the root folder, while the acquisition protocol in JSON format is available in the corresponding scan folder. All data can be accessed on OpenNeuro^27^. For each subject, unprocessed and processed images for structural and dMRI are provided, along with annotation maps and derivative results (Fig. 4). Demographic information (Table 2) was included for each participant in the data file (“participants.tsv”) as per the BIDS standard. The image quality reports are in the BIDS directory, where the information was name-matched with each scan’s name.

**Table 2:** Participant demographics.

Subject	Age	Sex	Type	Grading	Subject	Age	Sex	Type	Grading
Sub-01	35	M	Glioma	WHO III	Sub-11	34	M	Glioma	WHO III
Sub-02	35	M	Glioma	WHO II	Sub-12	43	F	Gray matter heterotopia	N/A
Sub-03	62	F	Meningioma	WHO I	Sub-13	28	M	Glioma	WHO II
Sub-04	64	F	Glioma	WHO IV	Sub-14	50	M	Glioma	WHO IV
Sub-05	37	M	Health control	N/A	Sub-15	31	F	Health control	N/A
Sub-06	53	F	Diffuse large B-cell lymphoma	N/A	Sub-16	53	F	Glioma	WHO II
Sub-07	54	F	Multiple sclerosis	N/A	Sub-17	46	M	Brain abscess	N/A
Sub-08	70	F	Multiple sclerosis	N/A	Sub-18	60	F	Glioma	WHO III
Sub-09	52	M	Diffuse large B-cell lymphoma	N/A	Sub-19	55	F	Glioma	WHO II
Sub-10	65	M	Glioma	WHO IV	Sub-20	48	F	Brain abscess	N/A

## Technical Validation

**Fig. 3:**
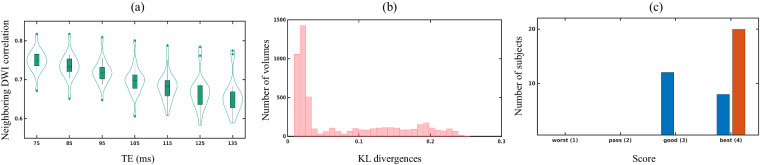
Plots for image quality metrics: (**a**) Neighboring DWI correlation (NDC), (**b**) KL divergences of rdMRI, and (**c**) quality score of structural images.
- We further used 3D Slicer module SlicerDiffusionQC (https://github.com/pnlbwh/SlicerDiffusionQC) for quality checking of diffusion-weighted MRI. It identifies bad gradients by comparing the distance of each gradient to a median line. The median line is obtained from Kullback–Leibler (KL) divergences between consecutive slices. Here, lower KL divergence values represent good data quality and vice-versa. In our data, none of the DWIs were identified with bad gradients (Fig. 3b).
- Each T1w/FLAIR image underwent thorough examination using the Structural MRI quality checking tool (https://github.com/pnlbwh/structuralQC) to ensure the quality and integrity of data. This QC algorithm first masks an input image with a foreground mask. Then, it slides a small cube throughout the volume, representing each cell with intensity histograms. After the histogram representation of the image, it is compared against a library of good and bad images and predicted as “pass” or “fail”. Figure 3c indicates none of the T1w/FLAIR images were identified as insufficient data.
- The rdMRI can be used to detect microscopic tissue features noninvasively, and relaxation time is linked to tissue biochemical composition. To obtain multi-dimensional imaging measures, mathematical modeling is combined with rdMRI data, allowing the measurement and mapping of microscopic tissue features. In a recent study^8,9^, joint moments of relaxation and diffusion were used to derive imaging measures. It was found that the estimated relaxation rate from *in-vivo* clinical data was reliable, as shown in Fig. 4. This supports the practical use of rdMRI in clinical settings.

To prompt the quality of our data, we assessed the quality of images using below common approaches:

- To assess the quality of each dMRI file, we utilized a measure of data quality known as the neighboring DWI Correlation (NDC), which can be found in the DSI Studio software (v2023.07.08) (https://dsi-studio.labsolver.org). We employed a comprehensive quality control (QC) procedure to meticulously examine each file, ensuring that image dimensions, resolution, DWI count, and NDC remained consistent across the board. The NDC measure summarizes the pairwise spatial correlation between each pair of dMRI volumes that sample the closest points in q-space. It computes a voxel-wise correlation coefficient between every two DWIs of the closest b-vector (multiplied by b-value). Then, it calculates the average of those coefficients across all voxels. Lower NDC values indicate reduced data quality due to noise and misalignment between dMRI volumes. Automatically computed quality measures for the entire image series, including the neighboring DWI Correlation, number of bad slices, and head motion summary statistics, are provided in the ‘derivatives/dwiqc/<subject>/<session>/dwi/<subject>_<session>_dwi.qc.txt file’. In our data, none of the dMRI voxels were identified as outliers (i.e., with a value greater than three times the mean), as the lowest NDC values were significantly higher than the suggested threshold of 0.6 (as shown in Fig. 3a).

**Fig. 4:**
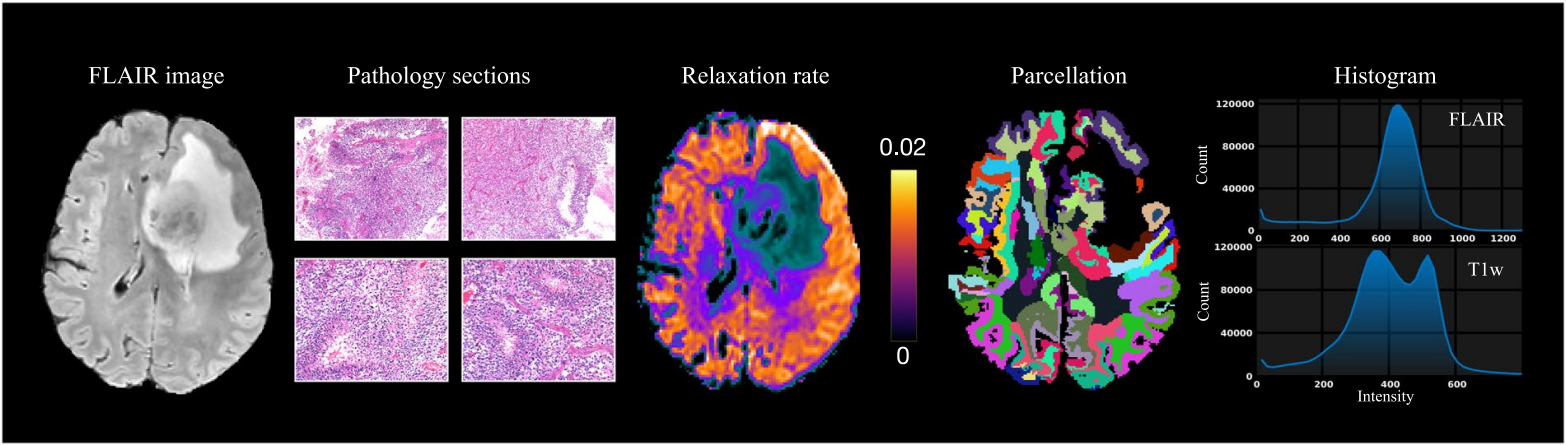
FLAIR imaging, pathology sections, and derivatives results include relaxation rate estimated by^9^, cortical parcellation generated by Freesurfer, and intensity distribution.

## Usage Notes

Our dedicated website will be regularly updated with additional data and analyses (https://github.com/dryewu/RDSI). We have created a forum topic where you can ask questions and get answers. This dataset is a valuable resource for neuroscientists who want to investigate behavioral models or anatomical systems that are not otherwise available, as well as for neuroimagers who wish to test their algorithms in combination with other resources available to researchers (for example, *ex-vivo* MAP dataset (https://www.drcmr.dk/map-datasets)). It will enable sharper phylogenetic investigations.

Relaxation-diffusion MRI is a technique that enables the characterization of intricate tissue microstructures and provides more detailed information about tissue properties than traditional diffusion MRI methods. This makes it particularly useful for studying evolutionary mechanisms and phylogenetics, as discussed in the following aspects.

Refinement of Evolutionary Mechanisms Modeling: Relaxation-diffusion MRI is a technique that provides highly detailed images of tissue microstructure, which can offer valuable insights into subtle changes that occur in tissue structure over long periods. By using this technique to examine changes in different species and relate them to known evolutionary histories, researchers can refine models of evolutionary mechanisms and gain a better understanding of the processes that drive evolution. Some studies have shown that transverse relaxation rate signals increase within higher-order association bundles during childhood and adolescence, suggesting an increase in myelination^28–32^.

Identifying Future Therapeutic Targets: Relaxation-diffusion MRI can detect changes in tissue microstructure that may indicate disease states or potential risks for certain diseases. By identifying areas of abnormality or potential risk, relaxation-diffusion dMRI can help to pinpoint possible therapeutic targets. For example, in neurodegenerative diseases like Alzheimer’s or Parkinson’s, relaxation-diffusion MRI can highlight areas of altered tissue microstructure in the brain that could potentially be targeted for treatment. A study by Ian F Harrison *et al*.^33^ suggests that MR relaxometry studies targeted to the standard and enlarged perivascular space may help detect dysfunction of perivascular fluid movement associated with aging and pathological conditions. Lewis *et al*.^34^ have explored the microvascular biomarkers using relaxation rate and found that inflammation is a crucial contributor to the tumor microenvironment and could be viewed as a therapeutic target in both vestibular schwannoma groups.

## Data Availability

Qualified researchers can access data from the current study (see the Participants section above for details). Scripts, supporting documents, and other information necessary to implement all aspects of data organization, preparation, and analysis using open-source software packages. Except for special instructions in the paper, all tools always use default parameters. No custom code was instructed.
